# The Antagonism of Therapeutic Agents

**Published:** 1878-10

**Authors:** 


THE ANTAGONISM OF THERAPEUTIC AGENTS, AND WHAT IT TEACHES. The essay to which was awarded the Fothergillian gold medal of the Medical Society of London, for 1878. By J. Milner Fothergill, M. D., Edin., member of the Royal College of Physicians, London, etc., etc. Philadelphia: Henry C. Lea. 1878.

This little volume of 160 pages gives the antagonism of certain toxic agents, from experiments on lower animals, and will be found a useful little book for the practicing physician.

